# Short form of the Changes in Outlook Questionnaire: Translation and validation of the Chinese version

**DOI:** 10.1186/1477-7525-10-41

**Published:** 2012-04-24

**Authors:** Yinyin Zang, Nigel C Hunt, Tom Cox, Stephen Joseph

**Affiliations:** 1 Institute of Work, Health and Organisations, University of Nottingham, Jubilee Campus, Wollaton Road, Nottingham, NG8 1BB, UK; 2 School of Sociology and Social Policy, University of Nottingham, University Park, Nottingham, NG7 2RD, UK

**Keywords:** Positive changes following adversity, Posttraumatic growth, Psychometric assessment

## Abstract

**Background:**

The Changes in Outlook Questionnaire (CiOQ) is a self-report instrument designed to measure both positive and negative changes following the experience of severely stressful events. Previous research has focused on the Western context. The aim of this study is to translate the short form of the measure (CiOQ-S) into simplified Chinese and examine its validity and reliability in a sample of Chinese earthquake survivors.

**Method:**

The English language version of the 10-item CiOQ was translated into simplified Chinese and completed along with other measures in a sample of earthquake survivors (n = 120). Statistical analyses were performed to explore the structure of the simplified Chinese version of CiOQ-S (CiOQ-SCS), its reliability and validity.

**Results:**

Principal components analysis (PCA) was conducted to test the structure of the CiOQ-SCS. The reliability and convergent validity were also assessed. The CiOQ-SCS demonstrated a similar factor structure to the English version, high internal consistency and convergent validity with measures of posttraumatic stress symptoms, anxiety and depression, coping and social support.

**Conclusion:**

The data are comparable to those reported for the original version of the instrument indicating that the CiOQ-SCS is a reliable and valid measure assessing positive and negative changes in the aftermath of adversity. However, the sampling method cannot permit us to know how representative our samples were of the earthquake survivor population.

## Background

Severely stressful and traumatic experiences can lead to both acute and chronic negative psychological change (e.g. [1-4]). However positive changes following adversity have also been recognised in philosophy, literature, and religion [5,6]. Against this backdrop, there is increasing empirical evidence showing that positive psychological changes can also result from the experience of events such as disasters [7], chronic illness [8], heart attacks [9], bone marrow transplants [10], HIV/AIDS [11], cancer[12], rape and sexual assault [13], military combat [14], bereavement [15], injury [16], recovery from substance addiction [17], and in the parents of children with disabilities [18].

The topic of growth through adversity has become the focus of much empirical [19,20] and theoretical work [21]. Through a process of struggling with adversity, changes may arise which lead the individual to reach a higher level of functioning than that which existed prior to the event. In the recent psychological literature, the positive changes that are observed following severely stressful events have been variously labelled perceived benefits [22], positive changes [23], posttraumatic growth [24], stress-related growth [25] and thriving [26].

A number of valuable psychological theories of positive changes have been proposed. Janoff-Bulman’s *shattered assumptions theory*[4] was developed prior to the establishment of this field but has provided the fundamental theoretical architecture for the two main theories of positive change, notably the *transformational model*[21] and the *organismic valuing theory*[27]. The *transformational model* provides a comprehensive theoretical description of posttraumatic growth. Individual characteristics, support and disclosure, and, more centrally, cognitive processing involving those structures threatened or nullified by the traumatic events, play important roles in this approach. Somewhat, by contrast, *organismic valuing theory* attempts to provide an account of positive changes rooted in humanistic psychology wherein post- traumatic stress is viewed as indicative of normal, natural cognitive processes that have the potential to generate positive change of such experiences. Several psychometric instruments have been developed to assess positive changes in the aftermath of adversity. These include: Changes in Outlook Questionnaire (CiOQ [23]); Posttraumatic Growth Inventory (PTGI [24]); Perceived Benefit Scales (PBS [22]); Stress-Related Growth Scale (SRGS [25]); and the Thriving Scale (TS [26]).

While some research has shown that posttraumatic growth is predictive of subsequent positive functioning, other research has failed to show such an effect. This has led to controversy over whether posttraumatic growth is necessarily beneficial including that of Park [28]. This is possibly due, in part, to measurement problems with current definitions of posttraumatic growth failing to distinguish between real growth and illusory growth. Self-reports of growth do not necessarily tally with other indicators as would be predicted. As such the use of retrospective self-report measures should be limited to those contexts in which other forms of assessment are unavailable [29]. In addition, much of the relevant research has been carried out in Western cultures [30] and it is not clear how such instruments might be applied elsewhere.

Although natural disasters are frequent in China, few studies have been published on growth following adversity in Chinese populations. To date only three studies [31-33] have reported posttraumatic growth in Chinese samples by using the Posttraumatic Growth Inventory (PTGI) which was developed based on a US sample. One study [31] uses the PTGI in Chinese cancer patients after exploring its factor structure. The other two studies use the PTGI and the IES-R to assess the prevalence and predictors of posttraumatic growth in a Chinese sample traumatised by an earthquake [32,33]. The present study examines a simplified Chinese version of the short form of Changes in Outlook Questionnaire [34], one of the most widely used self-report scales. The advantage of this measure is that it assesses both positive and negative schematic changes experienced following adversity and therefore offers a more complete picture of change.

The CiOQ [23] measures changes following the experience of severely stressful events [35]. The English language measure is composed of two subscales, an 11-item scale assessing positive changes (e.g. “I value my relationships much more now”), and a 15-item scale assessing negative changes (e.g., “I do not look forward to the future anymore”). The 26 items were originally generated by survivors of a shipping disaster in response to an open-ended question asking whether the disaster had changed their outlook on life in any way, either negatively or positively [36]. From respondents’ descriptions, a list of items purporting to represent positive and negative changes was drawn up. Joseph, Williams & Yule [23] reported satisfactory internal consistency for the positive and negative change scales (.83 and .90 respectively) and that those scales were not correlated (r = −.12). The measure has since been used in studies with a wide variety of participants following its experience of severely stressful situations, trauma and adversity, including people vicariously exposed to the 11 September 2001 terrorist attacks in the USA [37,38]; trauma therapists [39]; and members of general populations who have experienced adverse and traumatic events[35].

The 26-item CiOQ questionnaire is relatively long for both practitioners and others in need of a rapid means of assessment. Therefore, a short form CiOQ (CiOQ-S [34]), in English language, was developed by choosing the five highest loading items from the positive component and the five highest loading items from the negative component based of two-component structure. High internal consistency was reported for this version. Evidence to support the convergent and discriminant validity of the CiOQ-S was found with other measures of stress and trauma-related change. Compared with the CiOQ, the CiOQ-S enables researchers to gather data when there is only a limited amount of time available. This is very useful in many crisis situations.

The aim of this study is to translate the CiOQ-S into simplified Chinese and examine its factor structure, validity and reliability in Chinese earthquake survivors. It is worthy studying the relationship between positive changes and psychological variables as positive changes are assumed to make a difference in people’s lives by affecting levels of distress, well-being or other areas of mental health. Studies addressing the correlates of growth find that ruminative intrusion, social support, positive coping strategy are consistently associated with posttraumatic growth [19]. Correlations between posttraumatic growth and increased positive mental health, reduced negative mental health and better subjective physical health are found among individuals with cancer or HIV/AIDS [40]. Convergent validity was explored through the associations between the simplified Chinese version of the CiOQ-S (CiOQ-SCS) and measures of posttraumatic stress disorder (PTSD) symptoms, anxiety, depression, general health, coping style and social support.

## Method

### Participants

The data were collected from a mental health assessment programme for adults affected by the earthquake in Sichuan, China within the framework of an on-going recovery programme. The survey was carried out in December 2009 (19 months after the earthquake) among adults seeking assistance and their companies in Beichuan County. Beichuan was one of the areas nearly completely destroyed by the earthquake of 12th May, 2008. Almost all the buildings, including houses, working places, schools, and hospitals were destroyed. In Beichuan, 11522 people died and 9693 people were injured due to the earthquake [41]. People who had experienced the earthquake in Beichuan were recruited. The study was approved by the Ethical Committee of the University of Nottingham.

Due to a low literacy rate among the sample, an informed consent form was read to them. It contained a full explanation of the study objectives and explicit information about why their participation was being requested.

### Measures

The *Short Form of Changes in Outlook (CiOQ-S)* is a 10 items instrument consisting of 5 items measuring positive changes (CiOP-S: e.g., “I value my relationships much more now”), and 5 items measuring negative changes (CiON-S: e.g., “I don’t look forward to the future anymore”). Each item is rated on a six-point scale from strongly disagree (1) to strongly agree (6) so that there is a potential range of scores of 5 to 30 for both CiOP-S, and CiON-S. Higher scores indicate greater positive and negative changes, respectively. Joseph et al. [34], using a clinical sample, reported satisfactory properties of internal consistency reliability for both scales: .76 and .82, respectively. Higher scores on the negative change scale were associated with higher scores on the Posttraumatic Stress Disorder Symptom Scale (PSS): r = .61, p<.001. Positive changes were not related to scores on the PSS but were positively associated with the Posttraumatic Growth Inventory (PTGI): r = .46, p<.05.

*The Impact of Event Scale-Revised* (IES-R [42]) was used to assess the severity of posttraumatic stress disorder (PTSD) symptoms. This instrument is a self-report measure comprising 22 items and three subscales (intrusion, hyperarousal and avoidance), and scored on a 5-point Likert scale from not at all (0) to extremely (4). Cronbach alpha for the three subscales of the simplified Chinese IES-R have been reported as between 0.83-0.89 [43], providing good evidence that the simplified Chinese IES-R is a reliable and valid measure for assessing posttraumatic stress symptoms in a Chinese-speaking sample [44,45].

The *General Health Questionnaire-28* (GHQ-28 [46]) was used to assess the general mental health of the participants. This measure incorporates four subscales: somatic symptoms, anxiety and insomnia, social dysfunction, and severe depression. The simplified Chinese version of the GHQ-28 has been adopted widely and following its validation in Chinese [47] and with a reported Cronbach alpha of 0.92 with a sample of Chinese earthquake victims [48].

*The Hospital Anxiety and Depression Scale* (HADS) [49] was used to assess severity of depression and anxiety. The HADS is widely used as a brief self-rating instrument for both dimensional and categorical aspects of anxiety and depression. The internal consistency, as assessed by Cronbach’s alpha, is 0.76 for the depression subscale and 0.79 for the anxiety subscale in a sample of Chinese hospital in-patients [50].

The Multidimensional Scale of Perceived Social Support (MSPSS [51]) was used to measure social support. The scale is designed to assess perceptions of the adequacy of social support from three different sources: family, friends, and significant others. It consists of 12 items; each item is scored using a 7-point Likert scale ranging from 1 (strongly disagree) to7 (strongly agree). Adequate reliability and validity have been reported for a simplified Chinese version [52], with a Cronbach alpha of 0.89.

The *Simplified Coping Style Questionnaire* (SCSQ [53] is a simplified Chinese instrument measuring two dimensions of coping style: positive coping and negative coping. Active coping includes planning, thinking about solutions, and positive cognitive restructuring. Passive coping includes repression, behavioural disengagement, substance abuse and self-blame. There is high correlation among the 12 items for active coping styles (Cronbach alpha = 0.89) and the 8 items for passive coping styles. A Cronbach alpha of 0.78 was reported by Xie [53].

### Procedure

The 10 items of the CiOQ-S [34] were translated into simplified Chinese and then back-translated into English to confirm accuracy. This is a minimum requirement for the cross-cultural adaptation of established scales [54]. Disputed translations were discussed and resolved by 2 native Chinese speakers: one native Chinese psychologist and the first author. All were fluent in English and Mandarin. The CiOQ-SCS was read to 5 Beichuan local people. They were able to explore and discuss the items without assistance demonstrating correct understanding.

The translated scale (CiOQ-SCS) and the other measures (IES-R; GHQ-28; HADS; MSPSS; SCSQ) were administered orally by the first author, who is a native Mandarin speaker and qualified counsellor, in face-to-face interviews. Participants were informed that all scale items were focused on the earthquake as the trauma event to make sure that the latent psychological variables were associated with exposure to the earthquake. The duration of the interviews ranged from 30 to 60 min.

### Statistical analysis

The underlying structure of the CiOQ-SCS was explored through principal components analysis (PCA) using SPSS version 16.0, after first confirming that the data were suitable for factor analysis. This sample size (below 200) could not provide sufficient statistical power for confirmatory factor analysis [55]. The number of factors to be retained was guided by the theoretically driven factor structure proposed and three statistical decision rules described by Ferguson and Cox [56]: Kaiser's criterion (eigenvalues above 1), inspection of the scree plot and by the use of Horn's parallel analysis [57]. Parallel analysis was conducted using the software developed by Watkins [58].

Descriptive analyses including mean scores for each of the two subscales and characteristics of the participants were computed. The reliability of the two subscales was assessed using Cronbach alpha. Convergent validity was analysed -using correlations between the CiOQ-SCS and the measures of posttraumatic stress symptoms, mental health, anxiety, depression, social support and coping style.

## Results

### Principal components analysis (PCA)

Prior to performing PCA, the suitability of data for such modelling was assessed. The sample size (n = 120) of current study conformed to the minimum of 100 cases needed [56] and, additionally, the recommended 10 to 1 ratio of subjects to items [59]. Inspection of the correlation matrix revealed the presence of many coefficients of .30 and above also as recommended. The Kaiser-Meyer-Oklin coefficient was .77, and Bartlett’s test of Sphericity reached statistical significance, both supporting the suitability of the correlation matrix of modelling.

**Figure 1:**
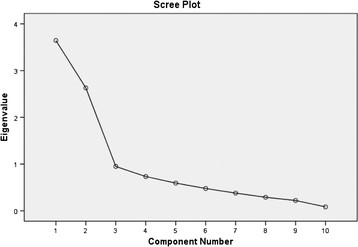
Scree plot showing the principal-components analysis with Varimax rotation of the 10 items.

PCA was used to extract the factors followed by orthogonal rotation using Varimax. PCA revealed the presence of two components with eigenvalues exceeding 1.00 (3.64, 2.63), explaining 36.44 % and 26.29 % of the variance respectively. An inspection of the scree plot [60] also suggested a 2 factor solution (see Figure 1). This was further supported by the results of Parallel Analysis, which showed only two components with eigenvalues exceeding the corresponding criterion values for a randomly generated data matrix of the same size (10 variables * 120 respondents). On the basis of this evidence, two factors were retained for further investigation.

The two-component solution explained a total of 62 % of the variance. There was a weak negative correlation between the two factors (r = −.12), but given the size of this correlation, the factors can be assumed to be independent. Varimax rotation was then performed (Table 1). The rotated solution revealed the presence of simple structure, with both components showing a number of strong loadings and all variables loading substantially on only one component. The interpretation of the two components was consistent with previous research on the CiOQ-S scale. Positive affect items loaded strongly on Component 1 and negative affect items loaded strongly on Component 2. The results of this analysis support the use of positive change items and the negative change items as separate scales, as suggested by the scale authors [34].

**Table 1:** Two solutions for the CiOQ-S with varimax rotation

**Items**	**Component1**	**Component2**	**Communalities**
4 CiOP-S	**0.87**	−0.10	0.77
5 CiOP-S	**0.87**	−0.13	0.77
10 CiOP-S	**0.84**	−0.01	0.70
6 CiOP-S	**0.78**	0.07	0.61
3 CiOP-S	**0.68**	−0.10	0.47
1 CiON-S	−0.18	**0.83**	0.71
2 CiON-S	−0.15	**0.83**	0.72
8 CiON-S	0.02	**0.76**	0.58
9 CiON-S	0.10	**0.69**	0.48
7 CiON-S	−0.08	**0.67**	0.45

### Descriptive analyses

The characteristics of the participants are presented in Table 2. The age range of the sample (N = 120) was 19 to 82 (mean = 56.90 ± 13.67). All participants had experienced the earthquake. Many were of low socioeconomic status: 63.3 % had no fixed income, and 36.7 % had income less than £300 per month (see Table 2). The means and SDs of CiOQ-SCS and other measure scores are presented in Table 3. Analyses were performed to reveal no difference between participants by age, gender, educational level, income, or extent of house damage. These variables were excluded from further analyses.

**Table 2:** The characteristics of the earthquake survivors (n = 120)

		**No.**	**%**
**Gender**			
	Male	40	33.3
	Female	80	66.7
**Marital status**			
	Single	6	5.0
	Married	98	81.7
	Divorced or widowed	16	13.3
**Education**			
	Primary or below	69	57.5
	Middle school	29	24.2
	High school	14	11.7
	Undergraduate	6	5.0
	Postgraduate	2	1.7
**Income**			
	No fixed income	76	63.3
	Less than £100/month	17	14.2
	£100-£300/month	27	22.5
**House damage**			
	No damage	5	4.2
	Slightly damaged	22	18.3
	Majorly damaged	43	35.8
	Totally ruined	50	41.7
**Loss**			
	None	6	5.0
	Below £500	33	27.5
	£500-£2000	18	15.0
	More than £2000	63	52.5
**Age group**			
	Mean(SD)	56.90(13.67)	
	Range	19-82	

**Table 3:** Means and SDs of measures

**Measures**	**Mean**	**SD**
CiOP-SCS	24.94	5.20
CiON-SCS	11.78	6.63
IES-R-intrusion	9.92	7.05
IES-R-avoidance	8.83	7.53
IES-R-hyperarousal	7.58	5.86
GHQ-28	7.60	7.79
HADS-depression	7.88	5.33
HADS-anxiety	5.86	4.27
MSPSS	61.68	12.05
SCSQ-active	23.23	6.78
SCSQ-passive	11.38	3.37

CiOP-SCS – Simplified Chinese version of Positive changes subscale of Short form of the Changes in Outlook Questionnaire.

CiON-SCS – Simplified Chinese version of Negative changes subscale of Short form of the Changes in Outlook Questionnaire.

### Reliability

The internal consistency of the scale was high with a Cronbach’s alpha of .87 for CiOP-SCS and .82 for CiON-SCS, which is comparable to the findings of Joseph et al. [34], where Cronbach’s alpha was .76 for CiOP-SCS and .82 for CiON-SCS.

### Convergent validity

Correlations between CiOQ-SCS scale and each of the psychopathology measures were computed (See Table 4). Scores on the CiON-SCS were positively associated with scores on IES-R intrusion, IES-R avoidance, IES-R hyperarousal, GHQ-28, SCSQ-passive, HADS-anxiety and HADS-depression, and negatively associated with scores on MSPSS and SCSQ-active. Scores on CiOP-SCS were positively associated with scores on the MSPSS and SCSQ-active, and negatively associated with HADS-depression. No statistically significant correlations were found with subscales of IES-R, GHQ-28, HADS-anxiety, and SCSQ-passive.

**Table 4:** Correlations with Measures

**Measure**	**IES-R**	**IES-R**	**IES-R**	**GHQ-28**	**HADS-**	**HADS-**	**MSPSS**	**SCSQ-**	**SCSQ-**
	**Intrusion**	**Avoidance**	**Arousal**		**Anxiety**	**Depression**		**Active**	**Passive**
CiOP-SCS	.067	.104	-.026	.006	-.167	-.193*	.278**	.215*	-.070
CiON-SCS	.420**	.470**	.530**	.458**	.400**	.534**	-.444**	-.435**	.223*

Note. *p<.05;**p<.01.

This null finding that no correlation between CiOP-SCS and intrusion was of interest as previous research has indicated an existed correlation between posttraumatic growth and intrusion [27,61]. The explanation for this is that the IES-R, used here, is not a sensitive enough measure of intrusion as it also includes items relating to sleep disturbance. To further explore this possibility the IES-R intrusion scale was split into a rumination–intrusion score and a separate sleep disturbance score (see [62]). Consistent with the explanation, scores on the CiOP-SCS were now associated with rumination–intrusion (r = 0.18, p<0.05) but not with sleep disturbance (r = 0.02), with CiON-SCS scores were partialled out to avoid any confounding effects.

## Discussion

This study validates the CiOQ-SCS for use by professionals in the field of traumatic stress working with Chinese population. The results confirmed the two-component structure of positive changes and negative changes, and correlations with other personality and social psychological variables showed that the CiOQ-SCS correlates significantly with other variables in predictable ways. Higher scores on the CiON-SCS were found to correlate with higher scores on measures of post-traumatic symptomatology and psychological distress. The results also showed the predictable correlations with anxiety, depression, lower social support, less active coping and greater passive coping. Higher scores on the CiOP-SCS were found to correlate with higher rumination-intrusion, lower depression, higher social support and active coping.

Previous work has shown that the negative schematic changes caused by trauma are related to posttraumatic stress [4]. These findings are supported here. In a study of the 911 terrorism attack in the USA, negative changes measured by the CiOQ were the most powerful predictors of heightened distress and diminished psychological well-being in both the short- and longer-term following the attack [37]. This could link to the view that profound challenges to basic assumptions about the self, others, and the world can be one of the most deleterious effects of traumatic experience [4]. These results also support the notion that positive changes are not simply the opposite of psychological distress but may be acting with such distress both independently, increasing positive growth, and in tandem, helping the person with meaning making after a traumatic event. This hypothesis is further supported by the significant correlation shown between positive changes and rumination-intrusion (IES-R). This finding is consistent with a previous study using the full CiOQ showing that positive changes were associated with the rumination-intrusion items but not the sleep-disturbance items of the IES [35]. Theoretical models of growth following adversity (see earlier [21,27]), propose that some degree of intrusion is necessary for growth to take place, as intrusions are indicative of cognitive processing which is a normal and necessary part of the adaptation process [61,63]. The finding that positive changes were not correlated with avoidance and hyperarousal is consistent with the full-CiOQ study [35], and also with a review by Zoellner and Maercker which reported no systematic relationship between posttraumatic growth and PTSD symptoms in most cross-sectional studies [64].

The negative correlation between positive change and depression found in this study is also consistent with previous research (e.g. [61,65,66]), and provides further evidence for the adaptive role of positive changes. The finding that no correlation existed between positive changes and anxiety is consistent with the review [19]. The null finding between positive changes and GHQ-28 also support the conclusion that no consistent relationship between positive change and general distress can be found in cross-sectional studies [64]

Although there is a wide range of age (19 to 82) in the sample, no significant difference across age groups was found. The low socioeconomic status and low educational level of the sample may have affected the results. Previous studies reported higher levels of education and income are associated with more adversarial growth [19,33].

As discussed in the background, there is an urgent need for cross-cultural studies on constructs such as growth following adversity that have been primarily investigated in Western populations. There are only three published studies exploring the posttraumatic growth in Chinese sample (see earlier [31-33]). The present study used an ethnically diverse sample to validate the Chinese version of the CiOQ-S. The inclusion of both positive and negative indicators of a traumatic experience precludes this study from the limitations of traditional trauma research. The study confirmed the 2-component structure of CiOQ-SCS and its reliability in a Chinese sample.

The results of this study revealed high levels of positive changes among Chinese earthquake survivors of low socioeconomic status. Although our sample was relatively small for such work, we have attempted to use a relevant sample rather than relying on a college student sample (see, e.g., [24,25]).

The authors acknowledge the limitations of this study. First, the sampling method used does not permit us to know how representative our samples were of the earthquake victim population. The sample contained more women than men, and this uneven sampling is not ideal for a psychometric study. However, this reflected the sampling typically found in research of this area [19] and of the survivor population. Second, the measures were presented verbally. People may like to be more positive when they are spoken to than when they are writing out their responses. However, given the level of literacy among the sample, there was little alternative. Third, although the short Chinese version of the CiOQ has reliable psychometric properties, posttraumatic growth is a theoretical concept that has been established within a Western cultural framework. Although at an abstract level the concept of posttraumatic growth appears cross-culturally valid, the operationalisation of the concept may serve to impose assumptions of a Western individualistic society [30] which emphasizes the individuation, uniqueness, and internal attributes of people. Chinese people are more relational and interdependent and emphasize the social context in comparison to their Western counterparts [67]. It is logical to expect that such differences should be relevant to the phenomenon of posttraumatic growth. Further research is needed to explore the nature of posttraumatic growth in Chinese or Eastern population.

There are several implications for future practice. First, this study is cross-sectional, and little is known on how positive changes at one point in time predict health-related outcomes at a later point. There is some evidence to suggest that positive changes predict distress and well-being in longitudinal studies [68,69]. CiOQ-S could be used in longitudinal studies assessing the relation between positive and negative changes and subsequent indices of psychological and physical functioning. Second, since positive changes are not simply the opposite psychological distress, investigations into therapeutic techniques to facilitate growth are now needed [70]. The CiOQ-S has been designed to be a simple and useful tool for clinical research to researchers and clinicians interested in both the alleviation of psychological distress and the facilitation of positive changes. Third, CiOQ-S might be used to collect data in many different crisis situations in short time.

## Conclusion

In summary, the findings from this validation study indicates that the simplified Chinese version of the CiOQ-S is a reliable and valid measure of positive changes and negative changes after adversity and now it can be used in studies of posttraumatic changes in Chinese people. The next step is to use the questionnaire in people traumatised by different events.

List of abbreviations: CiOQ, Changes in Outlook Questionnaire; CiOQ-S, Short form of the Changes in Outlook Questionnaire; CiOQ-SCS, Simplified Chinese version of Short form of the Changes in Outlook Questionnaire; CiOP-S, Positive changes subscale of Short form of the Changes in Outlook Questionnaire; CiON-S, Negative changes subscale of Short form of the Changes in Outlook Questionnaire; CiOP-SCS, Positive changes subscale of Simplified Chinese version of Short form of the Changes in Outlook Questionnaire; CiON-SCS, Negative changes subscale of Simplified Chinese version of Short form of the Changes in Outlook Questionnaire; PTSD, Post Traumatic Stress Disorder; IES-R, Impact of Event Scale-Revised; HADS, Hospital Anxiety and Depression Scale; PTGI, Posttraumatic Growth Inventory; PBS, Perceived Benefit Scales; SRGS, Stress-Related Growth Scale; TS, Thriving Scale; PCA, Principal components analysis; SCSQ, The Simplified Coping Style Questionnaire; GHQ-28, The General Health Questionnaire-28; MSPSS, The Multidimensional Scale of Perceived Social Support

## Competing interests

The authors declare that they have no competing interests.

## Authors’ contribution

YZ designed the study, translated the questionnaire, collected and analysed the data, and drafted the paper. NCH contributed to the study design, data analysis, questionnaire translation, and paper writing. TC and SJ contributed to the data analysis and paper writing. All authors read and approved the final manuscript.
